# Barriers to Healthcare for Latinx Autistic Children and Adolescents

**DOI:** 10.1007/s10803-023-06229-7

**Published:** 2024-01-17

**Authors:** Luke P Grosvenor, Ryan J Cohen, Nancy P Gordon, Maria L Massolo, Hilda J Cerros, Cathleen K. Yoshida, Jennifer L Ames, Lisa A. Croen

**Affiliations:** 1 Division of Research, Kaiser Permanente Northern California, Oakland, CA USA; 2 Columbia Medical School, New York, NY USA

**Keywords:** Healthcare Disparities, Autism Spectrum Disorder, Latino Children, Developmental Disabilities, Service Utilization

## Abstract

Purpose: To understand the ways in which autistic Latinx children experience disparities in diagnosis, healthcare, and receipt of specialty services. Methods: 417 individuals who identified as Latinx caregivers of autistic children who were members of the same integrated healthcare system in Northern California were surveyed. Responses were analyzed using the child’s insurance coverage (Government or Commercial) and caregiver’s primary language (Spanish or English). Results: Compared to the commercially-insured, government-insured participants accessed several services at a higher rate and were less likely to cite the high cost of co-pays as a barrier. Conclusion: There were no significant differences in service access by language status, but Spanish speakers were more likely to cite health literacy as a barrier to receiving care.

**Supplementary Information:**

The online version contains supplementary material available at 10.1007/s10803-023-06229-7.

Research on racial and ethnic disparities in ASD shows that Latinx children experience significant disparities in diagnosis, healthcare, and receipt of specialty services (Liptak et al., 2008; Magaña et al., 2012; Schieve et al., 2012). Studies have consistently reported a lower prevalence of ASD in the United States (U.S.) among Latinx children than non-Latinx Whites (Liptak et al., 2008; Maenner et al., 2020; Windham et al., 2011; Zuckerman et al., 2017), and Latinx children are more likely to be misdiagnosed (Durkin et al., 2017; Wiggins et al., 2020). Although past research showed that, on average, both Latinx and Black children were diagnosed with ASD later than White children (Mandell et al., 2002, 2005), a more recent study found that among children aged 3–4 years, the mean age of ASD diagnosis was comparable across racial, ethnic, and language groups (Jo H, 2015). Similarly, another recent study found that the time-lag between ASD diagnosis and engagement with behavioral intervention services did not differ by child race-ethnicity among children on Early Intensive Developmental and Behavioral Medical waivers (Yingling et al., 2018).

Once diagnosed with ASD, Latinx children have been less likely to receive specialty care such as behavioral therapy including applied behavioral analysis (ABA), occupational therapy, and social skills training (Magana et al., 2013; Magaña et al., 2016). For many Latinx families in the U.S., socio-cultural, language, structural, and financial factors may limit the ability of caregivers to access resources and healthcare services for their children with ASD (Denney, 2007). Recent studies estimate that half of Latinx children in the United States live in poor or near-poor households (Gennetian, 2019), and many do not have health insurance (Zuckerman et al., 2014). Type of insurance coverage (Medicaid versus private) also influences healthcare costs and utilization of health services related to ASD. For example, studies have reported that both total costs relating to all healthcare services, ASD-specific services and psychotropic medications as well as the total number of visits for each of speech, occupational, and behavior modification therapies were significantly higher among families on Medicaid compared to those with private insurance (Wang et al., 2013). A separate study comparing parent reports of access to services found no differences between Medicaid and privately insured groups, but that “waitlist” and “no coverage” were reported as a barrier for over 40% of the sample (Monz et al., 2019). These financial barriers are compounded by language barriers, difficulty navigating complex service systems, low levels of information about ASD and evidence-based treatments, high levels of mental health and disability stigma in the Latinx community, and negative experiences with healthcare providers (Rivera-Figueroa et al., 2022; Zuckerman et al., 2014). For example, compared to children of parents whose primary language was English, children of Spanish speaking parents were significantly less likely to have social skills and communications goals included in their individualized education plans (St Amant et al., 2018). Beyond spoken language, health literacy – the transferability of skills enabling access to and application of health information – is an important social determinant of health (Nutbeam & Lloyd, 2021). Differences in knowledge and understanding of what constitutes disability exist among Latinx communities, in part due to lack of cultural relevance within health communications and clinical care services, are key contributors to existing disparities (Garcia et al., 2019; Magana et al., 2013). Recurring themes of negative experiences with healthcare providers include caregivers feeling unheard when they expressed concerns about their child and receiving poor communication from providers, particularly during the early stages of their child’s diagnosis (Coffield et al., 2021).

The objective of this study was to identify barriers to and variation in the utilization of recommended assessment, diagnostic, and treatment services and access to health-related educational resources for ASD among a large and diverse population of Latinx children and adolescents who are members of the same integrated healthcare delivery system in Northern California. An improved understanding of the barriers to care for Latinx children with ASD may inform interventions for improving quality of care and the receipt of inclusive and culturally appropriate services for this growing population.

## Methods

### Study Setting

This survey study was conducted within the pediatric membership of Kaiser Permanente Northern California (KPNC). KPNC is a large, integrated, not-for-profit healthcare delivery system with 4.5 million insured children and adults who live in the San Francisco Bay area, Sacramento and South Bay areas, and surrounding counties. KPNC members are largely representative of the populations residing in the counties served by the health plan with respect to sociodemographic and clinical characteristics (Gordon, 2020). Regarding household income specifically, KPNC membership is somewhat more representative of individuals in median income categories than of those in the lowest and highest categories (Gordon & Lin, 2016; Krieger & Rowley, 1992).

### Study Sample

From the KPNC electronic health record (EHR), we identified all Hispanic/Latinx children aged 2–17 with a diagnosis of ASD who had at least 1 year of KPNC membership in the spring of 2018 (N = 3,262). To be included, an International Classification of Diseases, Ninth or Tenth Revision diagnosis of autism (ICD-9 299.0, 299.8, 299.9 or ICD-10 F84.0, F84.5, F84.8 F84.9) recorded in the child’s EHR on at least one occasion was required. For families with more than one child meeting these eligibility criteria in the same household, we selected the child with the most recent diagnosis. This sample was further classified into 4 subgroups based on information in the EHR about the primary caregiver’s preferred spoken language (Spanish or English) and whether the child was covered by Medicaid, Medicare, or state-subsidized insurance (Government) or by an employer group or individual insurance plan (Commercial). We randomly selected 300 children from each of the 4 subgroups (Spanish-Government (SG); Spanish-Commercial (SC); English-Government (EG); English-Commercial (EC)) and aimed to collect survey data for at least 100 children per subgroup. This sample size was chosen to provide sufficient power (80%) for detecting between-group differences in the proportions that reported experiencing barriers to healthcare. For dichotomous (yes/no) outcomes, we had sufficient power to detect a minimum difference in proportions of 0.10 (e.g., 0.20 vs. 0.30) when comparing across language or insurance coverage groups (e.g., for language: N > = 200 Spanish and N > = 200 English) and to detect a minimum difference in proportions of 0.16 (e.g., 0.20 vs. 0.36) when comparing across smaller language-insurance subgroups (e.g., N > = 100 in each of the SG, SC, EG and EC subgroups). For Likert scale outcomes, we had sufficient power to detect differences of at least 0.15 (e.g., 0.20 vs. 0.35) between two insurance-language subgroups (N > = 100 in each group) in one Likert scale category, assuming a corresponding change in the adjacent category.

### Data Collection

Data collection was conducted using a bilingual survey mailed to the caregiver of the target child. The postal mailing contained print study recruitment letters and questionnaires in Spanish and English and a pre-paid return envelope. Caregivers were instructed to complete the survey in the language with which they felt most comfortable. They were also informed that the survey could be completed over the telephone in their preferred language with the assistance of trained, bilingual research staff. The recruitment letter covered all the elements required by the KPNC IRB to waive written informed consent and HIPAA authorization to link survey responses with the child’s electronic health record (EHR) data. Caregivers were offered a $15 gift card as an incentive to complete the survey. Approximately three weeks after the mailing, a bilingual, trained research assistant called non-responders to remind them about the survey and the possibility of completing the survey over the telephone. Data collection started in May 2018 and continued through the end of the year.

### Survey Content

The Survey of Latino Parents about Use of Resources and Autism Services for Children was designed by the research team to capture relevant information about barriers to utilization of health services by adapting standard questions from the KPNC Member Health Survey (Gordon & Lin, 2016). It took approximately 20 min to complete on paper by self-administration or 30–60 min by phone interview. The survey collected information from caregivers about their family (caregiver & child) (1) sociodemographic characteristics, (2) social determinants of health, (3) caregiver literacy and health literacy, (4) past (any time) utilization of ASD-related assessment, diagnostic, intervention, and support services for the child, (5) past (3 months) barriers to use of ASD services, and (6) preferred methods for obtaining health-related information. The ASD service utilization, barriers to utilization, and health information preference data were collected using checklist questions, although caregivers were also given the opportunity to write-in other barriers to ASD care. Type of insurance coverage (government versus commercial) and caregiver spoken language preference (Spanish versus English) were ascertained from the child’s EHR. Some caregivers were reclassified by the study team into a different spoken language group based on information obtained through data collection. The survey was translated from English into Spanish by a bilingual research team member who is a certified translator, and the survey had an approximately 8th grade reading level. All participants were provided the option of completing the survey by phone. The English version of the survey is included in the Appendix.

### Survey Completion Rates

Surveys were completed for a total of 417 children (SG = 109, SC = 99, EG = 99, EC = 110). Approximately 20% of participants completed the survey as a phone interview and 80% returned the completed survey by mail. Spanish speakers were significantly more likely than English speakers to complete the survey by phone (SG = 22%, SC = 25%, EG = 7%, EC = 19%). A total of 18/417 (4.3%) of the final respondents were reclassified to the other language group by the study team based on the language used to complete the survey; 7 initially classified in the English language group completed the survey in Spanish, and 11 initially classified in the Spanish language group completed the survey in English and indicated in the survey that they spoke and read English well or very well.

### Statistical Analysis

We first evaluated differences in barriers to care across the two language groups (Spanish versus English) as well as across the two insurance coverage types (government versus commercial). For each language group, we also compared reported frequency of barriers by insurance type (e.g., among Spanish speakers, we compared barriers between government insured (SG) and commercially insured (SC)). Similarly, for each insurance type, we compared frequency of barriers between Spanish speakers and English speakers (e.g., among government insured, we compared Spanish speakers (SG) and English speakers (EG)). For all pairwise comparisons, we tested for significant differences between groups using chi-square tests and we report test statistics and *p*-values for all nominally significant (*p* < 0.05) differences in the Results. All analyses were performed using SAS v 9.4 (SAS Institute Inc., 2016).

## Results

### Characteristics of Study Population

Among the 417 children and adolescents with ASD, the mean age was 9.6 years (SD 4.3 years), and the male to female ratio was 5.1:1 (Table 1). Approximately 85% of caregivers who responded to the survey were the child’s mother, 23.2% had less than a high school education, 39.4% were not employed, 29.3% had an annual income below $35,000, 81.5% were married or in a committed relationship, and 82.3% lived in their own home with only their immediate family members. Sociodemographic characteristics varied significantly by primary spoken language and insurance type. Compared to caregivers whose primary language was English (N = 209), those whose primary language was Spanish (N = 208) were significantly more likely to have completed at most a high school education (78.2% vs. 22.6%; χ^2^ = 71.21, *p* < 0.001), to be unemployed (52.4% vs. 26.4%, χ^2^ = 29.28, *p* < 0.001), to have an annual income below $80,000 (91.3% vs. 68.4%, χ^2^ = 28.89, *p* < 0.001), to be married or in a committed relationship (89.4% vs. 73.6%, χ^2^ = 17.36, *p* < 0.001), and to live with their family only in their own home (90.9% vs. 73.7%, χ^2^ = 21.08, *p* < 0.001). Similar patterns were observed for those with government (N = 208) vs. commercial (N = 209) insurance coverage, respectively: a greater percentage reported completing at most a high school education (55.8% vs. 44.7%, χ^2^ = 4.86, *p* < 0.05), being unemployed (43.0% vs. 35.8%, χ^2^ = 6.94, *p* < 0.05), and having an annual income less than $35,000 (46.5% vs. 11.2%, χ^2^ = 54.80, *p* < 0.001). The proportion of caregivers in a committed relationship was lower among those with government versus commercial coverage (73.7% vs. 90.9%, χ^2^ = 19.93, *p* < 0.001).

**Table 1:** Characteristics of 417 study participants recruited from Kaiser Permanente Northern California who completed the Latino Barriers Survey

		Language Group			Insurance Group			Subgroup				
	**Total**	**Spanish**	**English**		**Government**	**Commercial**		**SG**	**SC**	**EG**	**EC**	
**Participants, N (%)**	417 (100)	208 (49.9)	209 (50.1)		208 (49.9)	209 (50.1)		109 (26.2)	99 (23.7)	99 (23.7)	110 (26.4)	
**Child age in years, Mean (SD)**	9.6 (4.3)	9.33 (4.29)	9.86 (4.29)		9.88 (4.16)	9.31 (4.42)		10.1 (4.3)	9.5 (4.3)	9.6 (4.1)	9.1 (4.5)	
**Child age**												
< 6	25.7	28.7	22.6		22.1	29.2		19.3	26.3	25.3	31.8	
6 to 11	42.7	41.2	44.2		43.8	41.6		44.0	44.4	43.4	39.1	
12 to 17	31.7	30.1	33.2		34.1	29.2		36.7	29.3	31.3	29.1	
**Child sex**												
Male	83.5	84.6	82.3		85.1	81.8		89.0	79.8	80.8	83.6	
Female	16.3	14.9	17.7		14.9	17.7		11.0	19.2	19.2	16.4	
Unknown	0.2	0.5	0.0		0.0	0.5		0.0	1.0	0.0	0.0	
**Role(s) of survey respondent(s)**												*^b,c^
Mother	85.4	82.2	88.5		88.5	82.3		82.6	81.8	95.0	82.7	
Father	8.4	7.2	9.6		3.9	12.9	*	4.6	10.1	3.0	15.5	
Mother & Father	5.8	10.1	1.4	*	7.7	3.8		12.8	7.1	2.0	0.9	
Other	0.5	0.5	0.5		0.0	1.0		0.0	1.0	0.0	0.9	
**Parent highest education**												*^b,c,d^
< High school	23.2	40.8	5.8	*	29.8	16.5	*	47.7	33.0	10.1	1.8	
High school	27.1	37.4	16.8	*	26.0	28.2		34.9	40.2	16.2	17.4	
Some college	25.6	10.2	40.9	*	28.0	25.2		8.3	12.4	45.5	36.7	
College graduate	24.2	11.7	36.5	*	18.3	30.1	*	9.2	14.4	28.3	44.0	
**Parent employment status**												*^c,d^
Not employed	39.4	52.4	26.4	*	43.0	35.8		56.5	48.0	28.3	24.8	
Works full-time	38.7	26.2	51.0	*	32.4	44.9	*	20.4	32.7	45.5	56.0	
Works part-time	22.0	21.4	22.6		24.6	19.3		23.2	19.4	26.3	19.3	
**Household income**				*								*^a,b,c,d^
< $35,000	29.3	32.6	26.4		46.5	11.2	*	45.7	16.7	47.3	7.0	
$35,000–80,000	49.9	58.7	42.0		46.0	53.9	*	52.1	66.7	39.8	44.0	
> $80,000	20.8	8.7	31.6	*	7.5	34.8	*	2.1	16.7	12.9	49.0	
**Marital status**												*^b^
Married or in committed relationship	81.5	89.4	73.6	*	73.0	90.0	*	85.3	93.9	59.2	86.4	
Separated/divorced	8.9	5.8	12.0	*	13.5	4.3		9.2	2.0	18.4	6.4	
Single/widowed	9.6	4.8	14.4	*	13.5	5.7		5.5	4.0	22.5	7.3	
**Current living situation of family**												*^c,d^
Lives alone in own home	82.3	90.9	73.7	*	80.3	84.2		90.8	90.9	68.7	78.2	
Shares home with others	15.8	8.7	23.0	*	17.8	13.9		9.2	8.1	27.3	19.1	
Other	1.9	0.5	3.4		1.9	1.9		0.0	1.0	4.0	2.7	

EC = English language-Commercial insurance; EG = English language-Government insurance; SC = Spanish language-Commercial insurance; SG = Spanish language-Government insurance; *p < 0.05; ^a^SG vs. SC; ^b^EG vs. EC; ^c^SG vs. EG; ^d^SC vs. EC

### ASD Services Used

**Fig. 1:**
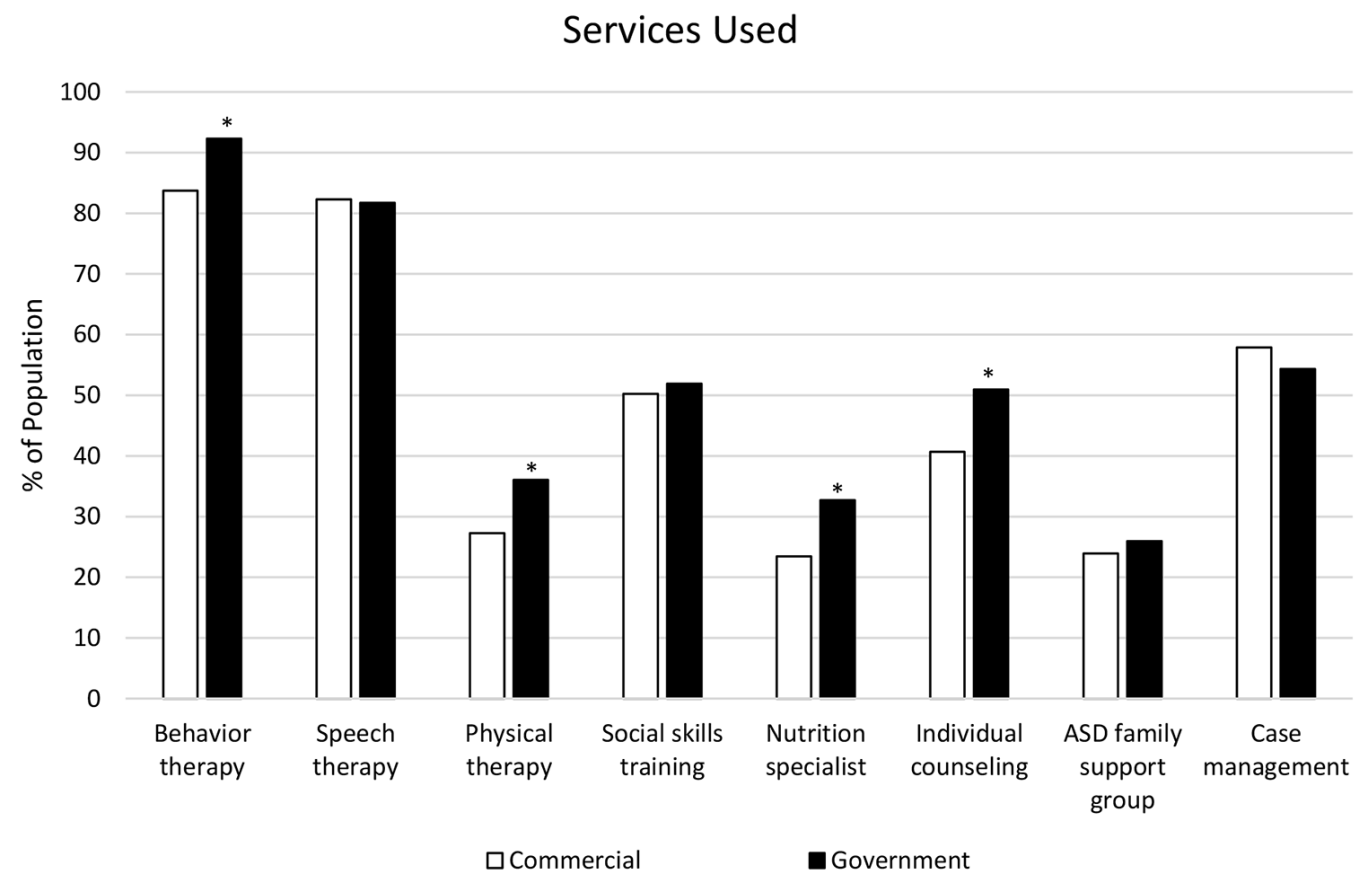
ASD service use by type of insurance. *indicates significance (*p* < 0.05)

The most frequently indicated ASD-related services used were behavior therapy (87.9%) and speech therapy (81.9%), followed by case management (55.7%), social skills training (50.6%), individual counseling/therapy (45.3%), physical therapy (30.8%), nutrition/feeding specialist consults (27.7%), and ASD family support groups (24.2%; Table 2). Caregivers in the government insured group (N = 208) were significantly more likely than those in the commercially insured group (N = 209) to indicate their child was using behavior therapy (92.3% vs. 83.5%, χ^2^ = 7.47, *p* < 0.01), physical therapy (35.4% vs. 26.2%, χ^2^ = 4.11, *p* < 0.05, difference driven largely by the SG subgroup), a nutrition/feeding specialist (32.7% vs. 22.7%, χ^2^ = 5.17, *p* < 0.05), and individual counseling/therapy (50.7% vs. 39.8%, χ^2^ = 4.97, *p* < 0.05; Fig. 1). Among commercially insured families, children ages 12–18 were significantly more likely than children less than 6 or between 6 and 11 years to use social skills training (67.2% versus 47.5% or 39.1%, respectively; χ^2^ = 11.53, *p* < 0.005; Table 2). A significantly higher percentage of children ages 12–18 also used individual counseling or therapy compared to the two younger age groups (less than 6 years and 6–11 years) for both the government (74.7% for 12–18 years vs. 28.3% (< 6 years) and 43.3% (6–11 years); χ^2^ = 27.51, *p* < 0.001) and commercially (52.5% vs. 28.3% (< 6 years) and 39.1% (6–11 years), χ^2^ = 7.31, *p* < 0.05) insured groups. There were no significant differences in ASD service use by caregiver language preference overall, but there were differences within each language group when comparing across child age groups. A significantly higher percentage of caregivers of children ages 12–18 in each language group reported using individual counseling or therapy, compared to the younger age groups (Spanish (N = 208): 64.7% of children ages 12–18, 40.7% of 6–11 and 39.1% of < 6 years, χ^2^ = 11.01, *p* < 0.005; English (N = 209): 64.5% of children ages 12–18, 41.9% of 6–11 and 20.0% of < 6 years, χ^2^ = 24.77, *p* < 0.001). Among only the English-speaking caregivers, a significantly higher percentage of children in the 12–18 year-old age group (71.0%) used social skills training compared to children ages less than 6 years or 6–11 years (48.3% and 44.2%, respectively; χ^2^ = 11.24, *p* < 0.005).

**Table 2:** Differences in ASD services used by study participants across preferred language, insurance coverage type, and age group

		Language Group			Insurance Group			Subgroup				
**Group**	Total	Spanish	English		Government	Commercial		SG	SC	EG	EC	
**N**	417	208	209		208	209		109	99	99	110	
**Behavior Therapy, %**	**87.9**	**85.4**	**90.4**		**92.3**	**83.5**	*	**91.7**	**78.1**	**92.9**	**88.2**	
< 6 years	92.5	87.0	96.7		95.7	90.0		95.2	80.0	96.0	97.1	
6–11 years	94.9	93.5	96.5		96.7	93.1		97.9	88.6	95.2	97.7	
12–18 years	74.6	73.1	76.2		84.5	62.7		82.5	59.3	87.1	65.6	
**Speech Therapy, %**	**81.9**	**78.7**	**85.1**		**81.7**	**82.1**		**81.7**	**75.5**	**81.8**	**88.1**	
< 6 years	84.9	82.6	86.7		84.8	85.0		85.7	80.0	84.0	88.6	
6–11 years	84.8	81.5	88.4		86.8	82.8		87.5	75.0	86.0	90.7	
12–18 years	75.6	72.5	79.0		73.2	78.3		72.5	72.4	74.2	83.9	
**Case Management, %**	**55.7**	**52.7**	**58.7**		**54.3**	**57.1**		**56.9**	**48.0**	**51.5**	**65.4**	
< 6 years	60.0	56.5	62.7		54.3	64.4		57.1	56.0	52.0	70.6	*^SC^
6–11 years	59.6	58.7	60.5		56.0	63.2		58.3	59.1	53.5	67.4	
12–18 years	46.9	42.0	52.5		52.1	40.7		55.0	24.1	48.4	56.7	
**Social Skills Training, %**	**50.6**	**47.8**	**53.4**		**51.5**	**49.8**	*	**50.9**	**44.3**	**52.0**	**54.5**	
< 6 years	45.7	42.2	48.3	*^E^	43.5	47.5	*^C^	42.9	41.7	44.0	51.4	
6–11 years	42.9	41.8	44.2		46.7	39.1		48.9	34.1	44.2	44.2	
12–18 years	64.9	59.4	71.0		62.9	67.2		57.5	62.1	70.0	71.9	
**Individual Counseling or Therapy, %**	**45.3**	**48.3**	**42.3**		**50.7**	**39.8**	*	**51.9**	**44.3**	**49.5**	**35.8**	*
< 6 years	28.3	39.1	20.0	*^S,E^	28.3	28.3	*^G,C^	33.3	44.0	24.0	17.1	*^SG,EG,EC^
6–11 years	41.2	40.7	41.9		43.3	39.1		38.3	43.2	48.8	34.9	
12–18 years	64.6	64.7	64.5		74.6	52.5		77.5	46.4	71.0	58.1	
**Physical Therapy, %**	**30.8**	**32.4**	**29.3**		**35.4**	**26.2**	*	**41.1**	**22.7**	**29.3**	**29.4**	*
< 6 years	26.4	30.4	23.3		32.6	21.7		33.3	28.0	32.0	17.1	
6–11 years	31.6	30.8	32.6		36.7	26.4		42.6	18.2	30.2	34.9	
12–18 years	33.3	35.8	30.6		35.7	30.5		43.6	25.0	25.8	35.5	
**Nutritionist, %**	**27.7**	**26.6**	**28.8**		**32.7**	**22.7**	*	**32.1**	**20.4**	**33.3**	**24.8**	
< 6 years	27.4	26.1	28.3		32.6	23.3		28.6	24.0	36.0	22.9	
6–11 years	28.7	27.2	30.2		31.9	25.3		31.3	22.7	32.6	27.9	
12–18 years	26.7	26.1	27.4		33.8	18.3		35.0	13.8	32.3	22.6	
**ASD Family Support Group, %**	**24.2**	**27.7**	**20.8**		**25.2**	**23.2**		**30.6**	**24.5**	**19.4**	**22.0**	
< 6 years	18.9	21.7	16.7		19.6	18.3		23.8	20.0	16.0	17.1	
6–11 years	24.4	29.7	18.8		24.7	24.1		36.2	22.7	11.9	25.6	
12–18 years	28.2	29.0	27.4		29.6	26.7		27.5	31.0	32.3	22.6	

EC = English language-Commercial insurance; EG = English language-Government insurance; SC = Spanish language-Commercial insurance; SG = Spanish language-Government insurance; *p < 0.05; ^S^Spanish; ^E^English; ^G^Government; ^C^Commercial; ^a^SG vs. SC; ^b^EG vs. EC; ^c^SG vs. EG; ^d^SC vs. EC; Nutritionist = Nutritionist/Dietician/Feeding Specialist

### Barriers to Receiving ASD Services

The percentages of caregivers indicating specific barriers to obtaining ASD services for their child are shown in Table 3. There were 4 categories of barriers: knowledge gaps, cost, English language proficiency/health literacy, and logistical issues. Knowledge gaps were the most frequently indicated barriers among the study sample (N = 417), with 29.7% reporting they did not know ASD services were available and 28.8% reporting that they did not know how to access these services (see Supplemental Table 1 for percentages indicating lack of awareness of specific ASD services that they had not used). Caregivers in the Spanish language group (N = 208) were significantly more likely than those in the English language group (N = 209) to report that they did not know services were available (34.6% vs. 24.9%, χ^2^ = 4.73, *p* < 0.05) or did not know how to access services (33.7% vs. 23.9%, χ^2^ = 4.82, *p* < 0.05). There were no significant differences between insurance groups with respect to awareness of services.

**Table 3:** Barriers to receiving recommended ASD services

		Language Group			Insurance Group			Subgroup				
	**Total**	**Spanish**	**English**		**Government**	**Commercial**		**SG**	**SC**	**EG**	**EC**	
**All participants, N (%)**	417 (100)	208 (49.9)	209 (50.1)		208 (49.9)	209 (50.1)		109 (52.4)	99 (47.6)	99 (47.6)	110 (52.4)	
**Knowledge gaps, %**												
Did not know services were available	29.7	34.6	24.9	*	28.9	30.6		33.0	36.4	24.2	25.5	
Did not know how to access services	28.8	33.7	23.9	*	26.9	30.6		32.1	35.4	21.2	26.4	
**Cost, %**												
High cost of co-pay	22.5	19.7	25.4		7.7	37.3	*	5.5	35.5	10.1	39.1	*^a,b^
Had trouble paying co-pays	26.1	24	28.2		11.5	40.7	*	11.0	38.4	12.1	42.7	*^a,b^
**English proficiency and health literacy, %**												
Does not understand/speak English well	23.3	46.2	0.5	*	24.5	22.0		45.9	46.5	1.0	0.0	
Has difficulty completing forms	10.3	14.4	6.2	*	10.6	10.1		13.8	15.2	7.1	5.5	
**Logistical issues, %**												
Put on a waiting list	23.5	22.6	24.4		26.4	20.6		25.7	19.2	27.3	21.8	
Had difficulty being home for child’s therapy	19.9	15.9	23.9	*	18.8	21.1		18.4	13.1	19.2	28.2	
High turnover rate in ABA agency	16.1	17.8	14.4		17.3	14.8		20.2	15.2	14.1	14.6	
Did not like someone being in home	10.3	9.1	11.5		10.6	10.1		11.0	7.1	10.1	12.7	
Had difficulty getting transportation	9.1	10.1	8.1		11.5	6.7		13.8	6.1	9.1	7.3	
Did not have space at home	8.9	12.5	5.3	*	11.5	6.2		17.4	7.1	5.1	5.5	
		Language Group			Insurance Group			Subgroup				
	**Total**	**Spanish**	**English**		**Government**	**Commercial**		**SG**	**SC**	**EG**	**EC**	
**Employed participants, N (%)**	251 (100)	98 (39.0)	153 (61.0)		208 (82.9)	133 (17.1)		47 (18.7)	51 (20.3)	71 (28.3)	82 (32.7)	
Had difficulty taking time off work for therapy, %	26.3	21.0	29.4		26.1	26.1		22.9	19.2	28.2	30.5	
Had difficulty being home for child’s therapy, %	22.3	19.0	24.8		21.0	23.9		20.8	17.3	21.1	28.1	

EC = English language-Commercial insurance; EG = English language-Government insurance; SC = Spanish language-Commercial insurance; SG = Spanish language-Government insurance; *p < 0.05; ^a^SG vs. SC; ^b^EG vs. EC; ^c^SG vs. EG; ^d^SC vs. EC

Over one-fourth (26.1%) of all caregivers had trouble paying for ASD services in the prior 3 months or indicated that high ASD service copays were barriers to care. Caregivers in the government insured group (N = 208) were less likely than those in the commercially insured group (N = 209) to report difficulty paying for ASD services (11.5% vs. 40.7%, χ^2^ = 45.82, *p* < 0.001) and high co-pays as a barrier (7.7% vs. 37.3%, χ^2^ = 52.41, *p* < 0.001). There were no significant differences between language groups with respect to cost barriers.

Nearly half (46.2%) of the Spanish language group reported not understanding or speaking English well compared to < 1% of the English language group. Overall, 10.3% of caregivers reported difficulty filling out intake forms or questionnaires, with those in the Spanish language group (N = 208) significantly more likely than English (N = 209) to report difficulty (14.4% vs. 6.2%, χ^2^ = 7.58, *p* < 0.01). There were no significant differences between insurance groups on this barrier.

The most frequently indicated logistical barrier to ASD services among all caregivers was being waitlisted for services (23.5%), followed by difficulty being home for child’s therapy (19.9%), high turnover rate at the ABA agency (16.1%), not liking to have someone in their home for so many hours (10.3%), difficulty getting transportation (9.1%), and lack of space at home for home-based therapy (8.9%). The Spanish language group (N = 208) was significantly less likely than the English language group (N = 209) to report difficulty being home for child’s therapy (15.9% vs. 23.9%, χ^2^ = 4.25, *p* < 0.05) but significantly more likely to report that they did not have enough space at home for in-home therapy (12.5% vs. 5.3%, χ^2^ = 6.75, *p* < 0.01). There were no other significant differences by language or insurance group regarding other logistical barriers. Among the 60% of all caregivers who were employed (N = 251), 26.3% had difficulty taking time off work and 22.3% had difficulty being home for their child’s therapy, with no significant differences between language or insurance groups.

### Social Risks

#### Low Educational Attainment and Health Literacy

The percentages of families with different social risks are summarized in Table 4. Overall, 23.2% of the study population did not complete high school. This low level of educational attainment was more prevalent in the Spanish (N = 208) versus English (N = 209) language group (40.8% vs. 5.8%, χ^2^ = 71.21, *p* < 0.001) and, within each language group, was more prevalent among those in the government versus commercially insured group (within Spanish: 47.7% government (SG) versus 33.0% commercial (SC), χ^2^ = 4.60, *p* < 0.05; within English: 10.1% government (EG) versus 1.8% commercial (EC), χ^2^ = 6.52, *p* < 0.05;). Among all caregivers surveyed, 23.5% reported low confidence in filling out medical forms by themselves and 32.0% reported having difficulty understanding written information. These health literacy challenges were significantly more common among the Spanish versus English language group (low confidence in filling out medical forms by themselves: 37.5% vs. 9.6%, χ^2^ = 45.24, *p* < 0.001; difficulty understanding written information: 48.3% vs. 15.8%, χ^2^ = 50.56, *p* < 0.001). While there were no significant differences in health literacy by insurance type, a higher proportion of the SG subgroup (N = 109) than the EG subgroup (N = 99) reported low confidence in filling out medical forms (43.1% vs. 12.1%, χ^2^ =_24.53, *p* < 0.001) and difficulty understanding written information (50.9% vs. 19.2%, χ^2^ = 22.64, *p* < 0.001).

**Table 4:** Prevalence of social risks among families with an autistic child or adolescent

		Language Group			Insurance Group			Subgroup				
	**Total**	**Spanish**	**English**		**Government**	**Commercial**		**SG**	**SC**	**EG**	**EC**	
**Participants, N (%)**	417 (100)	208 (49.9)	209 (50.1)		208 (49.9)	209 (50.1)		109 (52.4)	99 (47.6)	99 (47.6)	110 (52.4)	
**Educational attainment, %**												
Completed less than H.S. diploma	23.2	40.8	5.8	*	55.8	44.7	*	82.6	73.6	26.3	19.3	*^c,d^
**Health literacy, %**												
Low confidence completing forms by self^1^	23.5	37.5	9.6	*	28.4	18.7	*	43.1	31.3	12.1	7.3	*^c,d^
Difficulty understanding written health information^2^	32.0	15.8	48.3	*	35.8	28.2		50.9	45.5	19.2	12.7	*^c,d^
**Digital literacy and access, %**												
Unable to use the internet by self or with help	5.5	11.0	0.0	*	5.8	5.3		11.0	11.0	0.0	0.0	*^c,d^
Unable to use the internet by self	15.4	29.3	1.4	*	17.8	12.9		32.1	26.3	2.0	0.9	*^c,d^
Not signed up to use the health plan member portal	33.2	57.7	8.7	*	40.4	26.0	*	66.1	48.5	12.1	5.5	*^c,d^
**Social support, %**												
Never received unpaid help from family/friends	57.6	73.6	41.6	*	61.1	54.1		72.5	74.8	48.5	35.5	*^c,d^
Never/rarely receive enough emotional/educational support	46.5	53.4	39.7	*	43.3	49.8		49.5	57.6	36.4	42.7	*^d^
**Financial strains, %**												
Related to any healthcare or ASD services	31.4	33.5	29.3		19.2	43.5	*	18.4	41.8	20.2	45.5	*^a,b^
Trouble paying for medical/dental needs	12.4	12.4	12.4		12.8	12.1		13.5	11.3	12.1	12.7	
Trouble paying for ASD services	10.5	9.6	11.4		6.9	14.0	*	9.6	13.4	14.6	4.0	*^b^
Non-medical financial strains (food, housing, utilities, transportation, other)	36.9	40.7	33.2		43.3	30.6	*	33.9	32.3	53.5	29.1	*^b,c^
**Housing-related risks, %**												
Trouble paying for housing or heat/electricity in past 3 months, or concerned about lack of housing	37.9	36.1	39.7		45.2	30.6	*	39.5	32.3	51.5	29.1	*^b,c^
Had trouble paying for housing or heat/electricity	23.4	19.4	27.3		29.6	17.4	*	21.2	17.5	38.4	17.3	*^b^
Had concerns about lack of more permanent housing	6.5	7.7	5.3		9.6	3.4	*	11.9	3.0	7.1	3.6	*^a,b^
Had concerns about housing conditions (safety, noise, mold, etc.)	12.5	16.8	8.1	*	16.4	8.6	*	22.0	11.1	10.1	6.4	*^a,c^
**Food Insecurity, %**												
Had trouble paying for food or often worried that food would run out before there was money to buy	12.4	8.5	16.3	*	18.7	6.3	*	11.5	5.2	26.3	7.3	*^b,c^
Worried^3^ at least sometimes that food would run out before there was money to buy more	33.3	31.0	35.4		42.4	24.2	*	33.0	28.9	52.5	20.0	*^b,c^
**Transportation risks, %**												
Lack of transportation made it hard to get to medical appointments, ASD services, medication or medical supplies, do things related to everyday living, or trouble paying for transportation (past 3 months)	18.5	20.2	16.8		22.6	14.4	*	24.8	15.2	20.2	13.6	
Had trouble paying for transportation (past 3 months)	6.3	4.0	8.6		7.9	4.8		5.8	2.1	10.1	7.3	
**Other financial strains, %**												
Trouble paying for childcare	7.6	6.5	8.6		8.4	6.8		7.7	5.2	9.1	8.2	
Trouble paying for internet/phone	13.2	13.9	12.4		17.2	9.2	*	17.3	10.3	17.2	8.2	*^b^
Trouble paying for loans/debts	20.7	17.9	23.5		24.1	17.4		17.3	18.6	31.3	16.4	*^b,c^

EC = English language-Commercial insurance; EG = English language-Government insurance; SC = Spanish language-Commercial insurance; SG = Spanish language-Government insurance; *p < 0.05; ^a^SG vs. SC; ^b^EG vs. EC; ^c^SG vs. EG; ^d^SC vs. EC; ^1^Low confidence = Quite a bit or Extremely; ^2^Difficult = Sometimes/Often/Always; ^3^Worried = Sometimes/Often/Always

#### Digital Literacy and Access

Over 10% of caregivers in the Spanish language group (N = 208) were unable to use the internet to get information, fill out forms, or make payments even with help from someone. Nearly 30% were unable to perform these types of online tasks on their own, in contrast to just 1.4% of the English language group (N = 209; χ^2^ = 62.42, *p* < 0.001). Additionally, nearly 60% of caregivers in the Spanish language group were not signed up to use the health plan’s member portal, while a significantly lower percentage of caregivers in the English language group (8.7%; χ^2^ = 112.82, *p* < 0.001) were not signed up. The member portal enables members to communicate with their child’s healthcare team, view lab results, fill out forms, and schedule appointments.

### Social Support

Inadequate practical and emotional social support were issues for many of the caregivers surveyed, with 57.6% reporting that, in the last 3 months, they never received unpaid help from family or friends and 46.5% that they never or rarely got the amount of emotional or social support they needed. Social support challenges were significantly more common for the Spanish (N = 208) versus English (N = 209) language group, as indicated by never receiving unpaid help from family or friends in the past 3 months (73.6% vs. 41.6%, χ^2^ = 43.51, *p* < 0.001) or never or rarely receiving needed emotional or social support (53.4% vs. 39.7%, χ^2^ = 7.81, *p* < 0.01). There were no significant differences in social support between insurance group, but there were differences between language subgroups among commercially insured caregivers (N = 209). For example, compared to the EC subgroup (N = 110), a greater proportion of caregivers in the SC subgroup (N = 99) reported never receiving unpaid help from family and friends in the last 3 months (SC = 74.8%, EC = 35.5%; χ^2^ = 32.39, *p* < 0.001) and never or rarely receiving the emotional or social support they needed (SC = 57.6%, EC = 42.7%; χ^2^ = 4.60, *p* < 0.05).

### Financial Strains

In addition to the previously noted financial strains related to paying for ASD services, 12.4% of all caregivers surveyed experienced trouble paying for non-ASD related medical or dental needs in the previous 3 months, and 36.9% had difficulty paying for other basic needs, including food (12.4%), housing or utilities (23.4), transportation (6.5%), childcare (7.6%), internet access or mobile phone coverage (13.2%), and loans or debts (20.7%). Compared to the commercially insured group (N = 209), a greater proportion of the government insured group (N = 208) had difficulty paying for basic needs (45.2% vs. 30.6%, χ^2^ = 9.40, *p* < 0.005). Specifically, they had more difficulty paying for food (18.7% vs. 6.3%, χ^2^ = 5.83, *p* < 0.05), housing or utilities (29.6% vs. 17.4%, χ^2^ = 8.46, *p* < 0.005), and internet access or phone mobile coverage (17.2% vs. 9.2%, χ^2^ = 5.83, *p* < 0.05). The Spanish language group (N = 208) was significantly less likely than the English language group (N = 209) to have trouble paying for food (8.5% vs. 16.3%, χ^2^ = 5.74, *p* < 0.05, respectively).

Based on trouble paying for food or having often worried about not having enough money to buy food in the past 3 months, 12.4% of all families surveyed were classified as food insecure. Based on the additional Hunger as a Vital Sign risk of having sometimes worried about running out of food before having money to buy more, 33.3% were classified as at-risk for food insecurity. The percentages of families that were food insecure or at-risk for food insecurity were both higher in the government (N = 208) compared to commercially insured group (N = 209; food insecure: 18.7% vs. 6.3%, χ^2^ = 14.56, *p* < 0.001; at-risk: 42.4% vs. 24.2%, χ^2^ = 15.51, *p* < 0.001). A significantly greater proportion of the English-Government subgroup (N = 99) reported experiencing food insecurity (26.3%) or being at-risk for food insecurity (52.5%) compared to each of the English-Commercial (N = 110; 7.3% experienced insecurity, χ^2^ = 13.80, *p* < 0.001; 20.0% at-risk, χ^2^ = 24.10, *p* < 0.001) and Spanish-Government (N = 109; 11.5% experienced insecurity, χ^2^ = 7.23, *p* < 0.01; 33.0% at-risk: χ^2^ = 7.97, *p* < 0.005) subgroups.

Nearly 40% of all caregivers reported housing-related financial risk (trouble paying for housing or utilities, concern about the cost of housing or utilities, or concern about lack of more permanent housing), and 12.5% indicated concerns about their current living situation, including housing conditions, noise, and safety issues. Lack of or cost of transportation was a problem for 18.5% of all caregivers and was more prevalent in the government (N = 208) vs. commercially (N = 209) insured group (22.6% vs. 14.4%, χ^2^ = 4.70, *p* < 0.05).

### Preferred Methods for Getting Information and Advice About How to Manage Their Child’s ASD and Other Health Conditions

We grouped information modalities into three categories: person-based interactions, messages, and print/web-based media (Table 5). Of the person-based interactions, single or multi-session classes were most preferred (59.7%), followed by in-person counseling (51.4%), phone-based counseling (41.5%), talks held in community settings (33.8%), and video visits with doctors or patient educators (24.9%). The government insured group (N = 208) was significantly less likely to prefer video visits compared to the commercially insured group (N = 209; 20.4% vs. 29.3%, χ^2^ = 4.42, *p* < 0.05) and there were no significant differences by language group for person-based interactions. Greater than half of all caregivers (N = 417) were interested in four of the direct message modalities (57.7% regular mail, 55.8% messages sent through the patient portal, 55.1% links sent by regular email, 51.4% text messages) and 7.7% reported using Twitter feeds. The Spanish language group (N = 208) was more likely than the English language group (N = 209) to prefer regular mail (67.3% vs. 48.1%, χ^2^ = 15.72, *p* < 0.001) and Twitter feeds (11.5% vs. 3.9%, χ^2^ = 8.50, *p* < 0.005). For print and web-based materials, caregivers were most interested in obtaining information from websites (51.5%) and online videos (48.1%), with fewer participants reporting interest in podcasts (35.0%), fotonovelas (11.4%) and other print materials (35.7%). The Spanish language group was less interested in web-based information (43.3% vs. 59.7%, χ^2^ = 11.20, *p* < 0.001) and more interested in fotonovelas (18.8% vs. 3.9%, χ^2^ = 22.73, *p* < 0.001) than the English language group.

**Table 5:** Preferred methods of obtaining information and advice about how to manage ASD and other health conditions

		Language group			Insurance Group			Subgroups				
	**Total**	**Spanish**	**English**		**Government**	**Commercial**		**SG**	**SC**	**EG**	**EC**	
**Participants, N (%)**	417 (100)	208 (49.9)	209 (50.1)		208 (49.9)	209 (50.1)		109 (52.4)	99 (47.6)	99 (47.6)	110 (52.4)	
**Person-based interactions, %**												
Single or multi-session class	59.7	58.2	61.2		57.8	61.5		61.5	54.6	53.6	67.9	*^b^
In-person counseling session	51.4	51.0	51.9		53.9	49.0		56.9	44.4	50.5	53.2	
Phone counseling session	41.5	44.7	38.4		40.3	42.8		45.9	43.4	34.0	42.2	
Talks held in a community setting	33.8	35.6	32.0		33.5	34.1		37.6	33.3	28.9	34.9	
Video visit	24.9	21.6	28.2		20.4	29.3	*	22.9	20.2	17.5	37.6	*^b^
**Messages, %**												
Regular (postal) mail	57.7	67.3	48.1	*	56.8	58.7		64.2	70.7	48.5	47.7	
Secure patient portal messages	55.8	52.4	59.2		52.9	58.7		52.3	52.5	53.6	64.2	
Emailed links	55.1	53.9	56.3		53.4	56.7		54.1	53.5	52.6	59.6	
Text message	51.5	54.8	48.1		51.0	51.9		53.2	56.6	48.5	47.7	
Twitter feeds	7.7	11.5	3.9	*	7.8	7.7		11.0	12.1	4.1	3.7	
**Print and web-based materials, %**												
Information from websites	51.5	43.3	59.7	*	48.1	54.8		39.5	47.5	57.7	61.5	
Online videos	48.1	48.1	48.1		47.1	49.0		51.4	44.4	42.3	53.2	
Podcasts	35.0	38.0	32.0		33.5	36.5		37.6	38.4	28.9	34.9	
Print materials including fotonovelas	38.4	41.4	35.4		39.8	37.0		45.0	37.4	34.0	36.7	
Print materials only	35.8	37.5	34.0		36.9	34.6		40.4	34.3	33.0	34.9	
Fotonovelas only	11.4	18.8	3.9	*	10.7	12.0		16.5	21.2	4.1	3.7	

EC = English language-Commercial insurance; EG = English language-Government insurance; SC = Spanish language-Commercial insurance; SG = Spanish language-Government insurance; *p < 0.05; ^a^SG vs. SC; ^b^EG vs. EC; ^c^SG vs. EG; ^d^SC vs. EC

## Discussion

In this large Latinx population of families receiving healthcare from the same integrated health care system in northern California, socioeconomic status, health literacy, and social support were relatively low, and food insecurity and financial strains were relatively high. Overall use of ASD services was high, with over 80% of families within all language and insurance groups using behavioral and speech therapy. However, utilization of ASD services was higher for those with government insurance, and there were significantly greater language and health literacy barriers among those caregivers with a Spanish versus English language preference.

Our findings contrast with a previous study reporting no significant difference in the utilization of ASD services between government-insured and commercially insured participants in a United States-wide sample (Monz et al., 2019). However, they are consistent with an earlier survey of participants from 24 states in the U.S., which found that government insured participants accessed ASD services at a significantly higher rate than the commercially insured participants (Wang et al., 2013). These contrasting findings should be considered in the context of sample recruitment and survey methodology used, as well as secular trends in access to care. Monz et al. (2019) recruited families from a large, online research registry with a predominantly White, well-educated sample that was 100% English-speaking (SPARK Consortium, 2018) and collected parent-report information on access to care by asking about frequency or intensity of specific interventions. Wang et al. (2013) used insurance claims data, which represents the payers’ perspectives as opposed to the families’ and did not include individual-level sociodemographic information. Further, the claims data analyzed were from 2003 and many states have since passed bills mandating private insurance coverage of ASD. The current study addressed an existing gap in this literature, by describing differences in access to care across both insurance coverage type and spoken language, in addition to social determinants of health. Future research on ASD service initiation, continuation, and retention in relation to insurance type and type of healthcare delivery system would help clarify this literature.

We found that caregivers of the government insured children were significantly less likely than those in the commercially insured group to indicate having had trouble paying for ASD services or that high co-pays were a barrier to accessing ASD services. ASD services often require several visits per week or month, with each visit incurring a co-pay. The cost of specialty ASD services, in addition to a loss of parental productivity, has been reported as the greatest cause of financial burden for parents with children affected by ASD (Buescher et al., 2014). One possible explanation for our findings is that co-pays for ASD services may differ by insurance type. In California, health insurers are mandated to provide coverage for behavior health treatment for ASD for both commercially insured and government insured patients, but they may be required to waive the co-pay for ASD services for government insured patients (“S.B. 946,” 2011). A landmark study conducted from 1971 to 1982 showed that the presence of a co-pay reduced the use of nearly all health services (Brook, 2006). Another possible explanation for our findings is that, because some children who would not qualify for Medicaid by poverty would be able to qualify by disability through a Supplemental Security Income or a home-and-community-based waiver, the government insured sample of children could have greater overall functional severity (Semansky et al., 2011), and therefore be more likely to access ASD services. However, past research has found that Latinx children with greater levels of severity do not receive more services than children with less severe impairments, while severity does impact the number of services for White children (Magaña et al., 2016). More research is needed to understand the impact of functional severity on accessing ASD services in the Latinx community.

In our study, we found that 40% of caregivers in the Spanish language group had not completed high school and only 22% had formal education beyond high school. This lack of formal education, compounded by difficulty understanding oral and written English communications, may partially explain the large percentages of caregivers in the Spanish language group who indicated difficulty filling out forms and lack of awareness of ASD services or how to access those services as barriers to ASD services. An alternative explanation may be that many of the Spanish speaking caregivers may be recent immigrants who lack experience filling out traditional American medical forms. Additionally, participants in our study mentioned in interviews that they are unaware of resources because the healthcare system does not inform them of what is available. These barriers are consistent with past studies that show many members in the Latinx community cite language and lack of knowledge of how to access resources as barriers to receiving healthcare (Britigan et al., 2009).

Digital literacy may also be a barrier to information about ASD services and ability to enroll in them. In our study population, nearly 30% of caregivers in the Spanish language group reported that they were unable to use the internet to get information and perform online tasks like filling out forms and making payments on their own, while nearly all in the English language group were able to do so without help. Even when caregivers are aware of resources, inability to schedule appointments and complete intake forms on secure websites can lead to disparities in access to information and health care. Finally, lack of knowledge of ASD and ASD services may be compounded in the Latinx community by cultural stigma around disability and mental health issues (Chlebowski et al., 2018; Zuckerman et al., 2014, 2017).

Offering culturally preferred methods for exchanging information about ASD and ASD services is an important step towards improving pediatric ASD care for the Latinx population. Overall, caregivers in our survey preferred group classes and in-person counseling to phone counseling and video visits. The preference for in-person visits aligns with past work that found that members of the Latinx community tended to prefer direct contact for the purpose of research and data collection (Aponte-Rivera et al., 2014; Eakin et al., 2007; Miranda et al., 1996; Patrick et al., 1998). The government insured group was significantly less likely than the commercially insured group to prefer video visits. This may align with work showing that higher levels of poverty can be a barrier to telehealth accessibility (Saeed & Masters, 2021). With the explosion of telehealth tools following the COVID-19 pandemic, it is important for providers to understand patient and caregiver digital technological capabilities and preferences. Additionally, it will be essential for healthcare providers to address digital disparities more broadly to provide equitable ASD care. Future studies should address whether and how both the modality and frequency of services and interventions used by families may differ by spoken language, insurance type, and other social determinants.

One possible limitation of our study is that we only included families with children who had already received an ASD diagnosis. Studies have shown that there is underdiagnosis and delayed diagnosis in the Latinx community (Durkin et al., 2017; Liptak et al., 2008; Obeid et al., 2021; Schieve et al., 2012; Windham et al., 2011); therefore, our study findings are only generalizable to those families who are successful in getting their child diagnosed. Additionally, we did not have information for all children on how diagnoses were established, nor complete information on child’s age at time of ASD diagnosis or caregiver acculturation, including ethnic group and country of origin. These factors might be differentially associated with utilization of ASD services and barriers to care. Service utilization and barriers to accessing care may also differ according to functional severity of ASD, but we are unable to address this due to incomplete data on child ASD severity. A second limitation of recruiting our sample through KPNC is that all study participants had an established relationship with a healthcare provider and could potentially access a large network of services. Although government insured children make up 50% of the study sample, their experiences may still not be representative of those without any insurance, or those who access health services outside of KPNC. We cannot speculate about how those families may differ from our study population, and future research is needed to describe their experiences. Lastly, we may have encountered response bias due to caregiver relationships with the healthcare system that differentially impacted their willingness to respond. For example, caregivers who engaged with the system frequently may have been more likely to respond than those who had difficulty or encountered greater barriers. An improved understanding of these groups, particularly the non-responders who were unwilling or unable to engage in health services, warrants future research.

Several study strengths also deserve mention. Our comprehensive survey questionnaire enabled us to study many social determinants and barriers to healthcare and specifically to ASD services. Our study population was very large, and all participants belonged to the same health care delivery system and had the same set of services available to them. Additionally, ASD diagnoses were validated by a comprehensive standardized clinical assessment by autism experts for most study children.

In conclusion, the high cost of co-pays among commercially insured participants appears to be the most significant barrier in accessing ASD services in this Latinx community. Additionally, lower educational attainment, health literacy, and digital access were significant barriers for Spanish speakers. To better meet the needs of all members of the Latinx community who have children with ASD, it will be important to offer ASD materials in both Spanish and English and in both non-electronic and electronic formats and to ensure that Spanish-language ASD materials are accessible, culturally appropriate, and of the same quality as English materials. It will also be important to offer services that improve caregivers’ digital literacy overall and specifically with regard to use of the health plan and community agency websites and client portals.

## Electronic supplementary material

Below is the link to the electronic supplementary material.

Supplementary Material 1
