# Supplementary material for: Barriers to Healthcare for Latinx Autistic Children and Adolescents
Source: J Autism Dev Disord. 2024 Jan 17;55(2):605–19. doi: 10.1007/s10803-023-06229-7 (PMC11813993; doi:10.1007/s10803-023-06229-7)
Supplement: Supplementary file 1 — Supplementary Material 1 [file 10803_2023_6229_MOESM1_ESM.docx]

**Supplemental Table 1**. ASD services were not used due to lack of awareness or not being recommended by providers

|  |  | **Language Group** | |  | **Insurance Group** | |  | **Subgroup** | | | |
| --- | --- | --- | --- | --- | --- | --- | --- | --- | --- | --- | --- |
|  |  | **Spanish** | **English** |  | **Government** | **Commercial** |  | **SG** | **SC** | **EG** | **EC** |
| **Behavior therapy** | 50 | 30 | 20 |  | 16 | 34 |  | 10 | 21 | 7 | 13 |
| Didn't know about it | 20 (40.0%) | 13 (43.3%) | 7 (35.0%) |  | 5 (31.3%) | 15 (44.1%) |  | 3 (33.3%) | 10 (47.6%) | 2 (28.6%) | 5 (38.5%) |
| Not recommended | 20 (40.0%) | 14 (46.7%) | 6 (30.0%) |  | 8 (50.0%) | 12 (35.3%) |  | 6 (66.7%) | 8 (38.1%) | 2 (28.6%) | 4 (30.8%) |
|  |  |  |  |  |  |  |  |  |  |  |  |
| **Speech therapy** | 75 | 44 | 31 |  | 38 | 37 |  | 20 | 24 | 18 | 13 |
| Didn't know about it | 24 (32.0%) | 19 (43.2%) | 5 (16.1%) | * | 11 (29.0%) | 13 (35.1%) |  | 9 (45.0%) | 10 (41.7%) | 2 (11.1%) | 3 (23.1%) |
| Not recommended | 33 (44.0%) | 18 (40.9%) | 15 (48.4%) |  | 17 (44.7%) | 16 (43.2%) |  | 10 (50.0%) | 8 (33.3%) | 7 (38.9%) | 8 (61.5%) |
|  |  |  |  |  |  |  |  |  |  |  |  |
| **Physical therapy** | 285 | 138 | 147 |  | 133 | 152 |  | 63 | 75 | 70 | 77 |
| Didn't know about it | 64 (22.5%) | 39 (28.3%) | 25 (17.0%) | * | 31 (23.3%) | 33 (21.7%) |  | 17 (27.0%) | 22 (29.3%) | 14 (20.0%) | 11 (14.3%) |
| Not recommended | 190 (66.7%) | 85 (61.6%) | 105 (71.4%) |  | 90 67.7%) | 100 (65.8%) |  | 41 (65.1%) | 44 (58.7%) | 49 (70.0%) | 56 (72.7%) |
|  |  |  |  |  |  |  |  |  |  |  |  |
| **Social skills training** | 196 | 107 | 97 |  | 100 | 104 |  | 53 | 54 | 47 | 50 |
| Didn't know about it | 129 (63.2%) | 73 (68.2%) | 56 (57.7%) |  | 61 (61.0%) | 68 (65.4%) |  | 34 (64.2%) | 39 (72.2%) | 27 (57.5%) | 29 (58.0%) |
| Not recommended | 81 (39.7%) | 41 (38.3%) | 40 (41.2%) |  | 46 (46.0%) | 35 (33.7%) |  | 25 (47.2%) | 16 (29.6%) | 21 (44.7%) | 19 (38.0%) |
|  |  |  |  |  |  |  |  |  |  |  |  |
| **Nutrition/feeding specialist** | 300 | 152 | 148 |  | 140 | 160 |  | 74 | 78 | 66 | 82 |
| Didn't know about it | 101 (33.7%) | 54 (35.5%) | 47 (31.8%) |  | 46 (32.9%) | 55 (34.4%) |  | 27 (36.5%) | 27 (34.6%) | 19 (28.9%) | 28 (34.2%) |
| Not recommended | 175 (58.3%) | 79 (52.0%) | 96 (64.9%) |  | 81 (57.9%) | 94 (58.5%) |  | 37 (50.0%) | 42 (53.9%) | 44 (66.7%) | 52 (63.4%) |
|  |  |  |  |  |  |  |  |  |  |  |  |
| **Individual counseling/therapy** | 226 | 106 | 120 |  | 102 | 124 |  | 52 | 54 | 50 | 70 |
| Didn't know about it | 101 (44.7%) | 52 (49.1%) | 49 (40.8%) |  | 42 (41.2%) | 59 (47.6%) |  | 24 (46.2%) | 28 (51.9%) | 18 (36.0%) | 31 (44.3%) |
| Not recommended | 112 (49.6%) | 46 (43.4%) | 66 (55.0%) |  | 51 (50.0%) | 61 (49.2%) |  | 24 (46.2%) | 22 (40.7%) | 27 (54.0%) | 39 (55.7%) |
|  |  |  |  |  |  |  |  |  |  |  |  |
| **ASD family support group** | 313 | 149 | 164 |  | 154 | 159 |  | 75 | 74 | 79 | 85 |
| Didn't know about it | 174 (55.6%) | 85 (57.1%) | 89 (54.3%) |  | 82 (53.3%) | 92 (57.9%) |  | 41 (54.7%) | 44 (59.5%) | 41 (51.9%) | 48 (56.5%) |
| Not recommended | 94 (30.0%) | 38 (25.5%) | 56 (34.2%) |  | 49 (31.8%) | 45 (28.3%) |  | 23 (30.7%) | 15 (20.3%) | 26 (32.9%) | 30 (35.3%) |
|  |  |  |  |  |  |  |  |  |  |  |  |
| **Case management** | 217 | 98 | 85 |  | 95 | 88 |  | 47 | 51 | 48 | 37 |
| Didn't know about it | 107 (58.5%) | 60 (61.2%) | 47 (55.3%) |  | 53 (55.8%) | 54 (61.4%) |  | 27 (57.5%) | 33 (64.7%) | 26 (54.2%) | 21 (56.8%) |
| Not recommended | 67 (36.6%) | 34 (34.7%) | 33 (38.8%) |  | 39 (41.1%) | 28 (31.8%) |  | 20 (42.6%) | 14 (27.5%) | 19 (39.6%) | 14 (37.8%) |

EC = English language-Commercial insurance; EG = English language-Government insurance; SC = Spanish language-Commercial insurance; SG = Spanish language-Government insurance; *p<0.05
